# Supplementary figures and images for: Examination of distraction and discomfort caused by using glare monitors: a simultaneous electroencephalography and eye-tracking study
Source: PeerJ. 2023 Sep 15;11:e15992. doi: 10.7717/peerj.15992 (PMC10506577; doi:10.7717/peerj.15992)

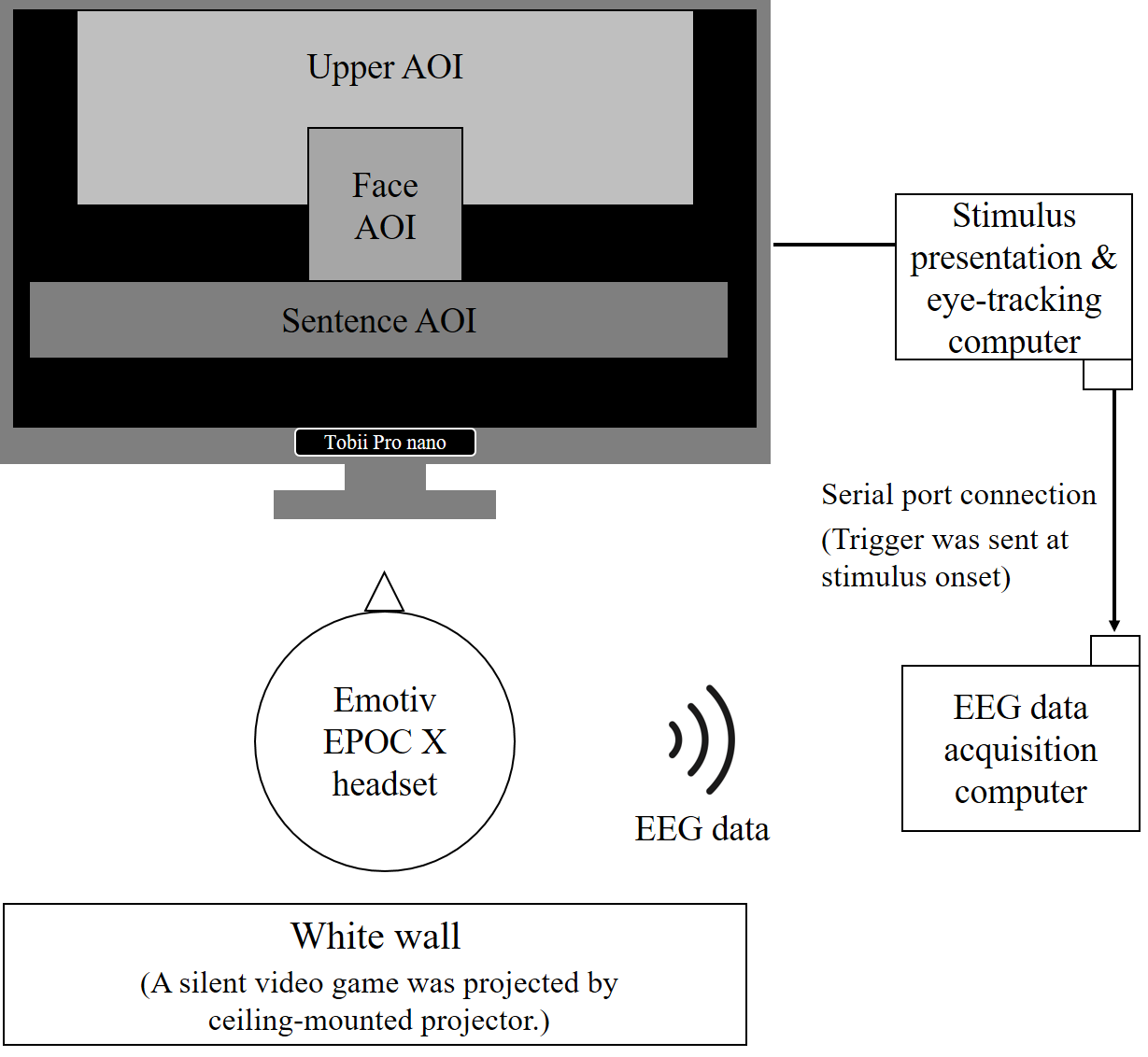

Supplement: Supplemental Information 2 — Sentence area of interest (AOI) was the place where the sentence was presented and its vicinity. Face AOI and upper AOI were the places where the participant’s face and the FPS video game, respectively, was reflected when using a glare monitor with a black background. Eye-tracking and EEG data were simultaneously recorded. [file peerj-11-15992-s002.png]
