# Supplementary material for: Examination of distraction and discomfort caused by using glare monitors: a simultaneous electroencephalography and eye-tracking study
Source: PeerJ. 2023 Sep 15;11:e15992. doi: 10.7717/peerj.15992 (PMC10506577; doi:10.7717/peerj.15992)
Supplement: Supplemental Information 3 — A glare monitor with a black background showed 57 fixations, which were more than those in the other conditions. Of these, 48 fixations were from two participants. [file peerj-11-15992-s003.docx]

**Supplemental Table S1:**

**Total number of fixations (i.e., summation of all participants) in the upper AOI from 0 to 5,000 ms.**

| **Background** | **White** | **Black** |
| --- | --- | --- |
| Glare monitor | 7 | 57 |
| Non-glare monitor | 11 | 2 |

In the glare monitor with a black background, there were 57 fixations, which were greater than the fixations in the other conditions. Of these, 48 fixations were from two participants. AOI, area of interest.
