# Supplementary material for: Examination of distraction and discomfort caused by using glare monitors: a simultaneous electroencephalography and eye-tracking study
Source: PeerJ. 2023 Sep 15;11:e15992. doi: 10.7717/peerj.15992 (PMC10506577; doi:10.7717/peerj.15992)
Supplement: Supplemental Information 4 — The results of an ANOVA using Greenhouse–Geisser correction with monitor and sentence/background color as within-participant factors indicated no significant main effect of monitor (F(1, 17) = 2.289, ηg2 = 0.008, p = 0.149), no significant main effect of sentence/background color (F(5, 85) = 2.719, ε = 0.59, ηg2 = 0.040, p = 0.065), and no significant interaction between monitor and sentence/background color (F(5, 85) = 0.852, ε = 0.59, ηg2 = 0.008, p = 0.483). [file peerj-11-15992-s004.docx]

**Supplemental Table S2:**

**Mean fixation count per sentence in the sentence AOI from 0 to 2,300 ms.**

| **Sentence** | | **Black** | **Blue** | **Yellow** | **White** | **Blue** | **Yellow** |
| --- | --- | --- | --- | --- | --- | --- | --- |
| **Background** | | **White** | | | **Black** | | |
| Glare monitor | Mean | 7.5 | 7.5 | 6.8 | 7.5 | 7.2 | 7.3 |
|  | SE | 0.3 | 0.3 | 0.2 | 0.3 | 0.2 | 0.2 |
| Non-glare monitor | Mean | 7.2 | 7.1 | 6.8 | 7.4 | 7.2 | 7.3 |
|  | SE | 0.3 | 0.4 | 0.2 | 0.1 | 0.2 | 0.3 |

The results of an analysis of variance using Greenhouse–Geisser correction with monitor and sentence/background color as within-participant factors indicated no significant main effect of monitor (F(1, 17) = 2.289, η_g_^2^ = 0.008, p = 0.149), no significant main effect of sentence/background color (F(5, 85) = 2.719, ε = 0.59, η_g_^2^ = 0.040, p = 0.065), and no significant interaction between monitor and sentence/background color (F(5, 85) = 0.852, ε = 0.59, η_g_^2^ = 0.008, p = 0.483). AOI, area of interest.
